# Automatic real-time uncertainty estimation for online measurements: a case study on water turbidity

**DOI:** 10.1007/s10661-019-7374-7

**Published:** 2019-04-02

**Authors:** Joonas Kahiluoto, Jukka Hirvonen, Teemu Näykki

**Affiliations:** 1 Environmental Measurement and Testing Laboratory, Finnish Environment Institute, Ultramariinikuja 4, 00430 Helsinki, Finland; 2 Environmental Measurement and Testing Laboratory, Finnish Environment Institute, Yliopistokatu 7, 80100 Joensuu, Finland

**Keywords:** Measurement uncertainty, Field measurement, The Nordtest approach, Water quality monitoring, Quality control, Turbidity

## Abstract

Continuous sensor measurements are becoming an important tool in environmental monitoring. However, the reliability of field measurements is still too often unknown, evaluated only through comparisons with laboratory methods or based on sometimes unrealistic information from the measuring device manufacturers. A water turbidity measurement system with automatic reference sample measurement and measurement uncertainty estimation was constructed and operated in laboratory conditions to test an approach that utilizes validation and quality control data for automatic measurement uncertainty estimation. Using validation and quality control data for measurement uncertainty estimation is a common practice in laboratories and, if applied to field measurements, could be a way to enhance the usability of field sensor measurements. The measurement system investigated performed replicate measurements of turbidity in river water and measured synthetic turbidity reference solutions at given intervals during the testing period. Measurement uncertainties were calculated for the results using AutoMUkit software and uncertainties were attached to appropriate results. The measurement results correlated well (*R*^2^ = 0.99) with laboratory results and the calculated measurement uncertainties were 0.8–2.1 formazin nephelometric units (FNU) (*k* = 2) for 1.2–5 FNU range and 11–27% (*k* = 2) for 5–40 FNU range. The measurement uncertainty estimation settings (such as measurement range selected and a number of replicates) provided by the user have a significant effect on the calculated measurement uncertainties. More research is needed especially on finding suitable measurement uncertainty estimation intervals for different field conditions. The approach presented is also applicable for other online measurements besides turbidity within limits set by available measurement devices and stable reference solutions. Potentially interesting areas of application could be the measurement of conductivity, pH, chemical oxygen demand (COD)/total organic carbon (TOC), or metals.

## Introduction

The need for reliable information about the environment is becoming more and more evident as mankind’s impact on the planet has significantly increased (Rockström et al. 2009). Measurements are needed to monitor and distinguish changes in the environment, to define if these changes are natural or an outcome of the human activity, and to evaluate the effects of policies which aim to keep our impact to the environment at an acceptable level (Elliott 2014). Long-term monitoring with comparable methods and known data quality is essential in utilizing the measurement data (Ellingsen et al. 2017). The chemical and physical state of natural waters is normally monitored by measuring different water quality parameters. Knowing the uncertainties of these measurements is critical as decisions are made based on the measurement results. Decisions made based on inaccurate data can have severe consequences. Surface water monitoring has traditionally been carried out using the combination of manual sampling and laboratory analysis, but there is a global trend shifting towards field measurements, remote sensing, and citizen science (Giles 2013; Nilssen et al. 2015; Dunbabin and Marques 2012; Conrad and Hilchey 2010). The quality of results produced in traditional environmental laboratories is ensured by using standardized methods, available guides and accreditation (Joint Committee for Guides in Metrology 2008; Eurachem/CITAC Guide CG 4 2012; International Organization for Standardization 2012; Magnusson et al. 2017; International Organization for Standardization 2017). For field measurements, the quality control procedures are not well enough established and the measurement uncertainties, including method and laboratory bias, are unknown or evaluated based on sometimes too ideal information provided by measuring device manufacturers (Björklöf et al. 2016a, b).

There is a need to improve the reliability, i.e., knowledge of measurement uncertainty, of these new monitoring methods in order to validate the produced data for decision making (European Commission 2009; Lewis and Edwards 2016). Continuous field measurements have a huge potential providing an unrivaled temporal resolution with better efficiency and lower costs per sample compared to sampling and laboratory analysis. Even classifying water bodies with conventional techniques can be questioned, because of the poor sampling density, when normal sampling intervals and laboratory analyses are used (Skeffington et al. 2015). Also, the greatest source of uncertainty is often caused by sampling, sample transport, and storage, which are at least partly eliminated in field measurements (Moser and Wegscheider 2001; Björklöf et al. 2016b). All of the necessary water quality parameters cannot be measured with online instruments and sensors at the moment (Näykki and Väisänen 2016), but technology is evolving and the list of parameters is growing all the time (Blomberg von der Geest et al. 2012).

Näykki et al. (2015) presented a way to apply the Nordtest method based on quality control and validation data (Magnusson et al. 2017; Hovind et al. 2011; International Organization for Standardization 2012) for “real-time” uncertainty estimations in online measurements, in which measurement uncertainty is broken down into within-laboratory reproducibility *u*_Rw_ and method/laboratory bias *u*_b_. These two components can be estimated from the data produced by the online measurement system, more specifically from routine sample replicate measurements and synthetic control sample measurements (Hovind et al. 2011). The idea of this approach for reliability estimation is fundamentally different when compared to a more traditional approach, where the reliability is defined through correlations with laboratory results. Comparisons with currently used methods, e.g., laboratory measurements, are required for detection of systematic differences between the methods, but for measurement uncertainty estimations, a continuous automated uncertainty calculation procedure is definitely able to reflect the current state of the measurement device better than an uncertainty value estimated once per year (or even lifetime) for an instrument. Requirements for the automated measurement system and automated data processing were laid out in the paper by Näykki et al. 2015. In this paper, it is reported how a remotely controlled automated measurement system and automated data processing system for uncertainty calculations was set up and tested in laboratory conditions. This will further clarify the practical issues arising from operating such a system and reveal possible problems with the approach and subjects for further research.

Water turbidity was selected as the research case for several reasons. First of all, the definition of turbidity according to ISO 7027-1 (International Organization for Standardization 2016) is “reduction of transparency of a liquid caused by the presence of undissolved matter,” which means that turbidity of water can be heterogeneous within a sample, as it is caused by undissolved matter with different particle sizes and densities (Horowitz 2013). Turbidity in itself is an important water quality parameter and in 2012, over 30,000 turbidity measurements from natural waters were carried out in Finnish laboratories (Näykki et al. 2014). Turbidity can also be used as a surrogate parameter for example in continuous suspended solid or nutrient monitoring when site- and instrument-specific relationships are well defined (Rymszewicz et al. 2017; Horowitz 2013; Caradot et al. 2015; Bilotta and Brazier 2008). Turbidity measurements are divided into two categories, nephelometry and turbidimetry, according to the measurement principle used. In nephelometry, diffused radiation in a 90° angle from the light source is measured whereas in turbidimetry attenuated radiation in a 0° degree angle is measured. Results gained with these two different principles have different units and are not comparable (Joannis et al. 2008). The measurement devices in this paper are nephelometric and the results are expressed in formazin nephelometric units (FNU). The primary reference solutions for turbidity measurements are formazin suspensions synthetized from hexamethylenetetramine (C_6_H_12_N_4_) and hydrazine sulfate (N_2_H_6_SO_4_) of which hydrazine sulfate is poisonous and may be carcinogenic (International Organization for Standardization 2016).

## Requirements for continuous field measurements with automated measurement uncertainty estimation

The requirements for automated measurement uncertainty estimations can be divided into two categories: hardware and software. The physical measurement station has to be able to perform replicate measurements from the sample water and also measure synthetic reference solutions at given intervals. The synthetic reference solutions have to be collected as waste in many cases, depending on the reference material used. For turbidity, this is the case because of the poisonous hydrazine sulfate. The software side is used to control the measurement station remotely, store the data into a database, and perform the uncertainty calculations with given settings from the produced measurement results. The reference solutions with certified reference values have to be distinguishable from routine samples and from other reference solutions with different certified values in a measurement series for the measurement uncertainty calculations. The calculated measurement uncertainties are then automatically attached to appropriate results. Transferring the results to different databases or presenting the results graphically can be implemented with suitable interfaces.

## Design and construction of the measurement station (hardware)

**Fig. 1:**
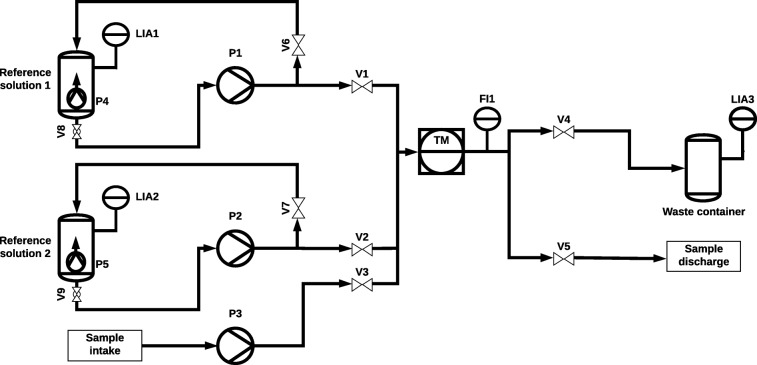
Measurement system process and instrumentation diagram (PID)

A water turbidity monitoring system (Fig. 1) was designed and constructed around a 1-m^3^ cylindrical tank simulating a river. Three pumps were installed into the tank to circulate and lift the water enabling the mixing of synthetic river waters with approximately known turbidities for testing purposes. The synthetic river water was prepared by mixing sediment from river Vantaa into tap water according to a defined correlation between sediment mass, water volume, and turbidity. The devices used in the measurement system (Fig. 1) are listed and described in Table 1.

**Table 1:** Device list

Device	Symbol in Fig. 1	Description
Reference solution containers 1–2	Reference solution 1–2	30 l conical
Waste container	Waste container	125 l cylindrical
Pumps for sample and reference solutions	P1-P3	Solinst 410
Reference solution mixing pumps	P4-P5	Biltema Art. 259750
Valves 1–7	V1-V7	Danfoss EV220B base with 24 VDC magnetic coils
Valves 8–9	V8-V9	Manual ball valve
Turbidimeter ABB	TM	ABB 7998 sensor with 4690 analyzer
Ultrasonic level indicator	LIA1-LIA2	DFRobot SEN0204
Waste container level indicator	LIA3	12″ eTape
Ultrasonic flow indicator	FI1	Cynergy3 UF08B100
Controlling computer	–	Raspberry Pi 3 Model B
I/O module	–	Arduino Mega
Relay card (× 2)	–	8× Songle SRD-05VDC-SL-C per card
4G modem	–	ZTE MF823
Power source	–	XP Power DNR480PS24-I
24 VDC/12 VDC converter	–	Biltema Art. 38-123

The liquid flows in the system were controlled with 12 relays to which the pumps and valves were connected to. Two different voltage levels were used (converted from the same power source) because the valves used are 24-V direct current (VDC) and pumps 12 VDC. Additional flow meter for volume flow information and control was installed, because the ABB turbidimeter specifications require a flow between 0.5 and 1.5 l/min, and the flow meter data can be used for quality control. Also, level indicators were installed to the reference solution containers and the waste container, to prevent running out of reference solutions and for avoiding waste overflow situations.

## Automation and remote management with Syke EnviCal Manager (software)

A cloud service based on open source solutions was programmed to control the measurement station. Syke EnviCal Manager consists of three modules: users, instrument platform, and data. The user module handles the user management and user rights. The instrument platform module enables sequential control of pumps, relays, and measurement devices as defined by the user and is used to perform sample water and synthetic control sample measurements automatically. The instrument platform also has features like real-time graphical monitoring, email alarms, and an alarm history log. The data module consists of a database, data analysis tools, and visualization tools. AutoMUkit software is implemented as a data analysis into the cloud for measurement uncertainty calculations. AutoMUkit calculates the measurement uncertainty for results within a specified time interval and concentration range(s) and combines the measurement uncertainty information to appropriate results either as absolute uncertainty (with the same unit as the measurement results) or relative uncertainty (percentage) with a specified coverage factor. Everything is controlled from a web user interface (UI) that also supports mobile devices.

## Automated uncertainty calculations

The automated “real-time” uncertainty calculation procedure applying the Nordtest approach presented by Magnusson et al. (2017) was introduced by Näykki et al. (2015) and is described in more detail in their paper. In brief, the method utilizes the standard deviation from routine sample replicate measurements (Eq. (1)) and the standard deviation from reference solution measurements (Eq. (2)) to estimate random error, i.e., within-laboratory reproducibility component (Eq. (3)).

1 $$ {u}_{r,\mathrm{range}}=\frac{\sum_{i=1}^{n_r}\left({c}_{(i)\max }-{c}_{(i)\min}\right)}{n_r\times d}\kern3.5em {u}_{r,\mathrm{range}\%}=\frac{\sum_{i=1}^{n_r}\left(100\%\times \frac{c_{(i)\max }-{c}_{(i)\min }}{\frac{c_{(i)\max }+{c}_{(i)\min }}{2}}\right)}{n_r\times d} $$

where *c*_max_ is the maximum and *c*_min_ is the minimum concentration in a replicate series, *n*_*r*_ is the number of replicate series, and *d* is a conversion factor from mean difference to the standard deviation (depends on the number of replicate series).

2 $$ {u}_{\mathrm{Rw},\mathrm{stand}}={S}_{\mathrm{Rw},\mathrm{stand}}\kern5.75em {u}_{\mathrm{Rw},\mathrm{stand}\%}=100\%\times \frac{S_{\mathrm{Rw},\mathrm{stand}}}{C_{\mathrm{avg}}} $$

*S*_Rw,stand_ is the standard deviation of control sample measurement results and *c*_avg_ the average of control sample measurement results.

3 $$ {u}_{\mathrm{Rw}}=\sqrt{u_{r,\mathrm{range}}^2+{u}_{\mathrm{Rw},\mathrm{stand}}^2}\kern3.25em {u}_{\mathrm{Rw}\%}=\sqrt{u_{r,\mathrm{range}\%}^2+{u}_{\mathrm{Rw},\mathrm{stand}\%}^2} $$

Bias is estimated from the difference between reference solution measurement results and certified reference value (Eq. (4)). The reference solution has to have a known certified reference value and a stated uncertainty for this value.

4 $$ {u}_{\mathrm{b}}=\sqrt{b^2+{\left(\frac{s_{\mathrm{b}}}{\sqrt{n}}\right)}^2+{u}_{\mathrm{cref}}^2} $$

where *b* is the difference between the control sample average and the actual reference value, *s*_b_ is the standard deviation of the control sample sensor measurements, *n* is the number of sensor measurement results of the control sample, and *u*_Cref_ is the standard uncertainty of the reference value.

5 $$ {u}_{\mathrm{c}}=\sqrt{u_{\mathrm{Rw}}^2+{u}_{\mathrm{b}}^2} $$

Combined standard uncertainty is then calculated from the reproducibility and bias components (Eq. (5)). Expanded uncertainty is calculated by multiplying the combined standard uncertainty with a coverage factor *k* (usually *k* = 2 for 95% confidence level). With this method, part of the repeatability component is included twice, but this is considered to be small compared to between-days variation (Magnusson et al. 2017). There are a lot of user-defined settings that can have a significant effect on the uncertainty estimations. These settings include dividing the estimation range depending on the behavior of the results as a function of the concentration, number of replicates, and sufficient time between different replicate sensor measurements. The settings include also the consideration of the amount of time (affecting the number of results) for which the measurement uncertainty is calculated for.

**Fig. 2:**
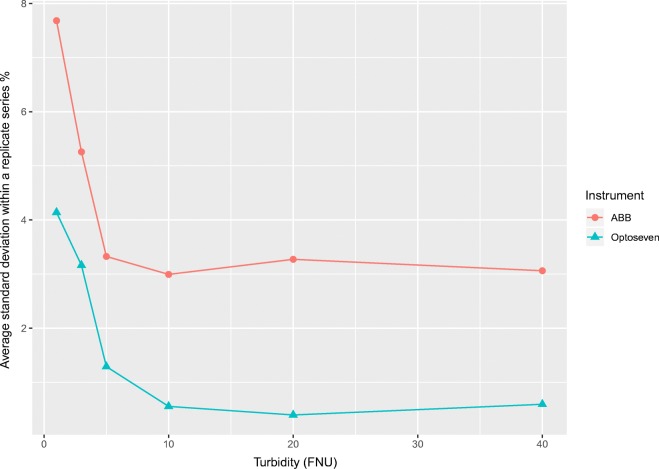
Standard deviation within replicate series as a function of turbidity with two different instruments

As presented in Fig. 2, the relative standard deviation within replicate series is stable only at concentrations higher than 5 FNU. Therefore, the measurement uncertainty estimation range should be divided into absolute and relative ranges at around 5 FNU. The uncertainty calculations should be performed in absolute units (FNU) from the limit of quantification up to 5 FNU and as relative for concentrations above 5 FNU (Magnusson et al. 2017; Kahiluoto 2017).

In laboratory measurements, the concept of a replicate measurement is well defined and includes the replication of all the analytical steps up to the result (Eurachem Guide 2011). For continuous measurements, there is no clear definition for a replicate measurement and data can be collected at very short intervals down to 1 ms for this system. Replicates should be measured from the same sample and at intervals where the random variation within the sample and the measurement system noise are representative. In this work, an approach where the interval is estimated based on sample flow rate and the internal volume of the measurement system was selected. This way, the samples can be seen as separate sub-samples, but the time for the sampled water body to change is minimized. In practice for our measurement system with around 1-l/min flow rate and a 0.15-dm^3^ cuvette volume, this lead to 10-s intervals between replicate measurements.

Another important aspect to consider is the time period for which the measurement uncertainty is calculated for. There has to be enough data for the uncertainty estimation, which would favor a long period of data collection, but on the other hand, the measurement uncertainty will likely change with time as biofouling and instrument drift affect the results. Biofouling is an acute problem in open measurement systems (sondes etc.), possibly affecting the results only after several days, but the problem also exists in flow-through systems (Delauney et al. 2010). Biofouling is also highly site-dependent, environmental condition–dependent, and temperature-dependent, which complicates the situation. The reference solutions can serve as a way to evaluate the drift caused by biofouling provided that the reference solutions can be reproducibly measured during the operating period. The authors of Näykki et al. (2015) suggest that at least 30 measurement results over a period of 10 days are to be used for the estimation, which can serve as a starting point together with Hovind et al. 2011, which states that at least 5% of measurements in laboratories should be quality control measurements. In ISO 11352 (2012), at least six certified reference material measurement batches are recommended for the estimation of bias.

## Stability and mixing of formazin reference solutions

One week of autonomous operation can be considered a minimum requirement for a cost-effective measurement station and hence, the minimum stability requirement for the reference solutions. According to the manufacturer, the stability of formazin reference solutions is 1 month, when the concentration is between 20 and 400 FNU and only 12–24 h, when the concentration is 2–20 FNU (Sadar 2003). The stability of formazin solutions diluted from 4000 FNU stock solution (Thermo Scientific, Orion AC45FZ) was studied in Hach 2100 AN IS sample cuvettes (30 ml) and for selected turbidities in the actual high-density polyethylene (HDPE) reference solution containers. The laboratory measurements were conducted with a Hach 2100 AN IS turbidimeter (calibrated with a HACH Stablcal® calibration kit before the experiments) according to ISO 7027-1 (2016). Measurement uncertainty (*k* = 2) for laboratory measurements was estimated to be 0.12 FNU in the concentration range 0–5 FNU and 8.9% in the concentration range 5–40 FNU, calculated with MUkit software according to Magnusson et al. (2017). In the cuvette scale test, seven solutions per concentration level (1, 5, and 20 FNU) were prepared and monitored for alteration of turbidity values during a 33-day test period. The turbidities of the solutions remained adequately stable for 1 to 3 weeks depending on the turbidity, with a higher turbidity leading to a better stability.

**Fig. 3:**
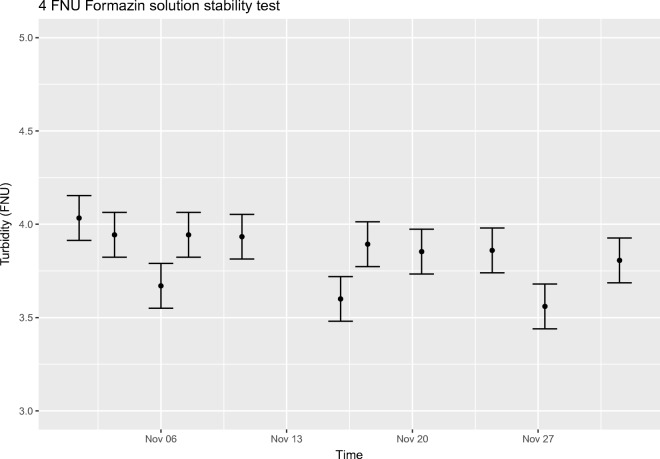
4-FNU formazin solution stability test results

**Fig. 4:**
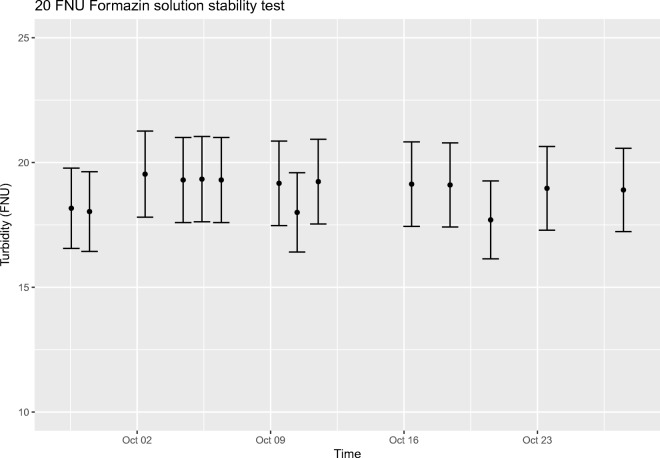
20-FNU formazin solution stability test results

Because of the possible effects caused by differences in volume, container material, and exposure to air, the turbidities of 4 FNU and 20 FNU formazin solutions were also studied with a 10-l batch volume in the actual reference solution containers. The solutions were thoroughly mixed by manually shaking and tilting the container and with a mixer attached to a drill to achieve homogeneity before sampling. The samples were collected with a pipette from around six random locations from the top half of the container and turbidities were measured immediately. The results show that the stability of the diluted formazin solutions fulfill the minimum requirement of 1-week stability, with no detectable decline in turbidity for the 20 FNU solution and only a slight decrease for the 4 FNU solution during the 4-week test period. The results are shown below in Figs. 3 and 4, where the measurement uncertainties presented do not include uncertainty caused by sampling.

## Results from the simulation experiments

**Fig. 5:**
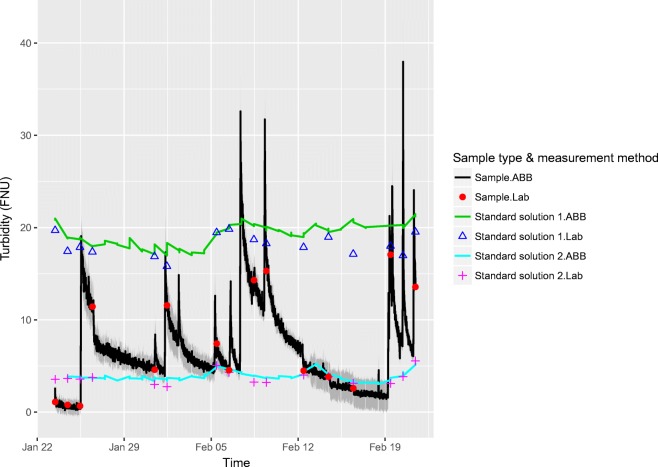
Simulation experiment results. Black line represents measurement results and gray area around the results describes the calculated measurement uncertainty (*k =* 2) for the online turbidity sensor

An experiment simulating the intended use of the measurement station was set up in laboratory conditions. A 1-m^3^ tank with pumps circulating synthetic river water prepared from sediment and tap water simulated a flowing water body. Sediment and tap water were added and the bottom of the tank was stirred manually multiple times during the experiment. The measurement station was programmed to measure 5 replicate measurements with 10-s intervals from the synthetic river water (referred to as sample) once per hour. The reference solutions were measured once every 24 h (three results saved with 1-s intervals). The uncertainties of the diluted reference solutions used in the experiments were estimated with GUM workbench pro (version 2.4) to be 2.3% for the 20 FNU reference solution and 0.1 FNU for the 4 FNU solution (*k* = 2). The measurement uncertainty calculations were performed weekly (four calculation runs in total), in order to include a satisfactory number of reference solution results. Two-week and 1-month calculation intervals were also tested for the same data set. A total of three reference solution measurement results had to be deleted due to detected ABB instrument malfunction during the test. The test results are presented graphically in Fig. 5 and the calculated measurement uncertainties are tabulated in Table 2.

**Table 2:** Measurement uncertainty calculation results

Calculation interval	Number of replicate series in 0–5-FNU range	Reproducibility within-laboratory *u*_Rw_ (FNU)	Method and laboratory bias *u*_b_ (FNU)	Expanded measurement uncertainty for the 0–5-FNU range expressed in FNU (*k =* 2)	Number of replicate series in 5–40-FNU range	Reproducibility within-laboratory *u*_Rw%_ (%)	Method and laboratory bias *u*_b_ (%)	Expanded measurement uncertainty for the 5–40-FNU range expressed in % (*k =* 2)
1-week calculation intervals								
23 Jan–30 Jan 2018	48	0.22	0.34	0.81	103	7.4	6.7	19.9
30 Jan–6 Feb 2018	50	0.57	0.15	1.18	111	6.2	11.7	26.4
6 Feb–13 Feb 2018	23	0.41	0.088	0.83	138	5.0	2.7	11.2
13 Feb–22 Feb 2018	143	0.79	0.16	1.62	52	5.2	2.9	11.9
2-week calculation intervals								
23 Jan–7 Feb 2018	104	0.49	0.15	1.02	230	8.1	7.9	22.5
7 Feb–22 Feb 2018	160	0.65	0.13	1.33	174	5.1	2.6	11.3
1-month calculation interval								
23 Jan–22 Feb 2018	264	0.57	0.12	1.2	404	7.9	4.9	18.6

**Fig. 6:**
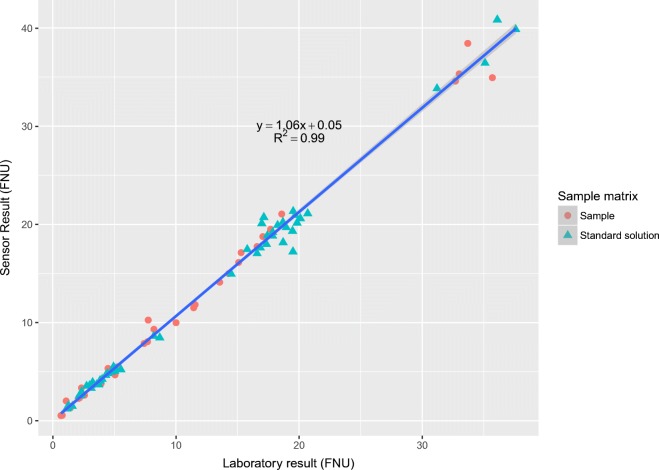
Laboratory results compared with sensor results

A total of 45 laboratory samples were taken for comparison at the same time as the measurement station performed measurements. The river water laboratory samples were taken with a pump through a separate line from the same spot in the tank as the measurement system intake (± 10 cm). Reference solution laboratory samples were taken with a pipette straight from the containers. The laboratory samples were analyzed immediately with a Hach 2100 AN IS turbidimeter. The 45 laboratory measurements from this experiment complemented by 48 comparison measurements from earlier experiments are presented in Fig. 6. The laboratory results and sensor results show a strong correlation across the measurement range (*R*^2^ = 0.99).

The instrument drift during the experiment was studied by measuring a dry secondary reference after the primary calibration of the instrument before the experiment and measuring the same dry reference again after the experiment. The secondary reference yielded a result of 1.333 FNU before the experiment and 1.404 FNU after the experiment resulting in a 5% drift. This can also be seen in Fig. 5, where the difference between the laboratory and sensor measurement results for reference solution 1 seemed to increase towards the end of the experiment.

The limit of quantification (LOQ) was studied from the average standard deviation within replicate measurement sets measured from ~ 1 FNU river water. Twenty-seven measurements were performed over a period of 2 days and the LOQ was calculated multiplying the average standard deviation within replicate measurement sets (0.12 FNU) by 10 leading to LOQ = 1.2 FNU.

**Fig. 7:**
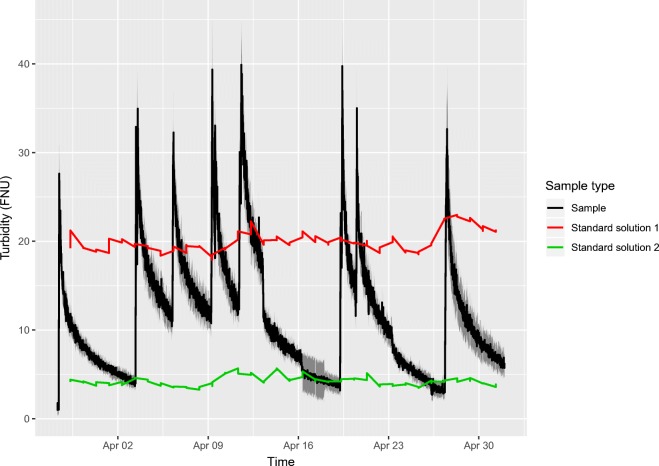
Results of the second continuous measurement test. Black line represents measurement results and gray area around the results describes the calculated measurement uncertainty (*k =* 2) for the online turbidity sensor

A second similar experiment without laboratory sample comparison was conducted between 28 March and 2 May 2018. Results of the second simulation experiment are presented with 1-week calculation intervals in Table 3 and Fig. 7.

**Table 3:** Measurement uncertainty calculation results for simulation experiment 2

Calculation interval	Number of replicate series in 0–5-FNU range	Reproducibility within-laboratory *u*_Rw_ (FNU)	Method and laboratory bias *u*_b_ (FNU)	Expanded measurement uncertainty for the 0–5-FNU range expressed in FNU (*k =* 2)	Number of replicate series in 5–40-FNU range	Reproducibility within-laboratory *u*_Rw%_ (%)	Method and laboratory bias *u*_b_ (%)	Expanded measurement uncertainty for the 5–40-FNU range expressed in % (*k =* 2)
28 Mar–4 Apr 2018	33	0.33	0.13	0.71	121	2.4	5.3	11.7
4 Apr–11 Apr 2018	0	–	–	–	161	5.1	5	14.3
11 Apr–18 Apr 2018	32	0.59	0.85	2.08	129	5.6	2.5	12.3
18 Apr–25 Apr 2018	29	0.42	0.17	0.91	132	5.8	2.2	12.3
25 Apr–2 Apr 2018	51	0.41	0.11	0.84	109	8.4	6.5	21.3

## Results and discussion

According to the test results, it is evident that the results of the online measurement system compare well with the laboratory measurements (*R*^2^ = 0.99). The estimated expanded measurement uncertainties (*k* = 2) are close to the recommended ± 20% for turbidities over 5 FNU (being ± 19% if calculated for the whole month and even less in the second experiment), but the recommended limit of quantification 0.5 FNU and expanded measurement uncertainty of ± 0.2 FNU for low measurement range (0.5–1 FNU) was not reached with this system (Näykki and Väisänen 2016).

The different measurement uncertainty calculation intervals presented in Table 2 demonstrate the temporal dimension of measurement uncertainty estimation for continuous measurement systems. If data from a long period of time is used for the estimation, in this case, 1 month, which can still be considered as a relatively short period, the resulting measurement uncertainty ends up being an average over the time period. When shorter evaluation periods are used, the estimated measurement uncertainty reflects the current performance of the system better, but the estimation becomes more vulnerable to outliers. An example of this can be seen in the second week of Table 2 (30 January–6 February), where the reference solution batch used for the relative uncertainty estimation had a lower than expected concentration (both sensor and laboratory results), leading to a high *u*_b_ and therefore a high measurement uncertainty. With the 1-month calculation interval, the effect of this one batch of reference solution to the calculated measurement uncertainty is significantly smaller. The same effect could be caused also for example by a short period of challenging measuring conditions or rapid biofouling between maintenances. There is a significant variation in both *u*_Rw_ and *u*_b_ which would infer that the performance of the measurement device (measurement uncertainty) depends on the properties of the measured samples (*u*_Rw_) and the state of the measuring system (*u*_b_). The highest measurement uncertainties were calculated for weeks (30 January–6 February and 11 April–18 April) where *u*_b_ component was high due to differences between the reference solution batches. This highlights the importance of trueness and homogeneity of the used reference solutions.

Based on laboratory standards and this limited amount of data produced in laboratory conditions from a single parameter (turbidity) with only one matrix and one measurement device, the authors suggest the following measurement uncertainty calculation settings for possible further research:

- The results used for a measurement uncertainty estimation should cover a minimum 1-week time period
- The number of synthetic control sample measurement results used for a measurement uncertainty estimation should be at least 6 for each estimation range (International Organization for Standardization 2012)
- The number of replicate sample measurements used for a measurement uncertainty estimation should be at least 60 for each estimation range (Magnusson et al. 2018)
- The measurement uncertainty estimation could be performed with the same interval as the reference solutions are measured, e.g., daily (autonomous mode)

During the study, the AutoMUkit software had to be run manually to calculate measurement uncertainties for past measurement results. In the future, an autonomous mode will be implemented, which enables calculation of the measurement uncertainty on a set interval, e.g., daily using a defined quantity of historical data, e.g., 100–200 results during 1 or 2 weeks. AutoMUkit will then attach the calculated measurement uncertainties to the future measurement results until the next uncertainty calculation is performed. This way, the most recent measurement uncertainty value represents the current state of the system and the measurement uncertainty estimation is close to real time.

The procedure, described by Näykki et al. (2015) which was tested in this paper, is not limited to only turbidity measurements and it can be applied for uncertainty estimations in any automated continuous measurements in which routine sample replicate measurements and reference material measurements are performed sequentially. The same uncertainty estimation procedure could in theory also be utilized with online gas analyzers. The real limitations are set by the availability of suitable reference materials and measurement devices. Most of the field measurement devices and sensors are not so called flow-through model, which is required because of the need to measure synthetic reference solutions, but this problem can be solved simply by installing the sondes or sensors into a flow-through cell. A more severe problem can be caused by the availability of stable reference solutions and reference solution mixing, because after all, a dilute reference solution should retain a stable reference value throughout the container volume for long enough time periods.

## Conclusions

In the future, where continuous measurements will most likely have a significantly larger role in environmental monitoring, this approach should be studied in field conditions and with different measurements to gain knowledge about suitable parameters and calculation settings. For all applications, such a heavy quality control system is not needed, but this paper demonstrates that it is possible to have an automated quality control system for continuous field measurements. The system is capable of producing measurement results with higher quality and traceability compared to current best available commercial technologies, considering the additional information on data quality, but at the cost of higher maintenance requirements. Also, special care should be taken to ensure the quality of reference solution measurements as failures to accurately and reproducibly measure the synthetic reference solutions will cause overestimated measurement uncertainties.
